# Development of Gram Stain Scoring System Based on Pro-Inflammatory Cytokines in the Sheep Model for Testing Toxicity of Vaginal Products

**DOI:** 10.3389/frph.2021.714798

**Published:** 2021-12-06

**Authors:** Kathleen L. Vincent, Aaron L. Miller, Carrie Maxwell, Nicola Richardson-Harman, Cynthia O'Neill, Lauren N. Dawson, Timothy Madsen, Cattlena Walker, Glenn Swartz, Richard B. Pyles

**Affiliations:** ^1^Department of Obstetrics and Gynecology, The University of Texas Medical Branch at Galveston, Galveston, TX, United States; ^2^Department of Pediatrics, The University of Texas Medical Branch at Galveston, Galveston, TX, United States; ^3^Department of Microbiology and Immunology, The University of Texas Medical Branch at Galveston, Galveston, TX, United States; ^4^Alpha StatConsult LLC., Damascus, MD, United States; ^5^Advanced Bioscience Laboratories, Inc., Rockville, MD, United States; ^6^Office of Clinical Research, The University of Texas Medical Branch at Galveston, Galveston, TX, United States; ^7^Sinclair Research Center, Auxvasse, MO, United States

**Keywords:** ovine sheep vaginal model, toxicity testing, intravaginal drug delivery, vaginal microbiome, proinflammatory cytokines, Gram stain scoring system

## Abstract

**Background:** Development of safe, effective products to prevent the sexual transmission of HIV remains a priority. Prior to clinical testing, the products must undergo strict safety evaluations to avoid mucosal drug toxicity, inflammation, and vaginal microbiome (VMB) shifts. Based on the Food and Drug Administration (FDA) guidance, we designed a study to measure the inflammatory markers and VMB changes after intravaginal treatment with products that have been associated with toxicity, with the objective to develop a Gram stain slide scoring system, similar to Nugent scoring, correlated with the proinflammatory cytokines in sheep.

**Methods:** Non-pregnant Dorset ewes (*n* = 34) were randomized to receive 5 ml intravaginal 4% nonoxynol-9 (N9) contraceptive gel, positive control (0.2% benzalkonium chloride), placebo control [hydroxethyl cellulose (HEC)], or no application daily for 10 days, with 11-day post-treatment follow-up. The vaginal swabs were collected for the cytokines, VMB, and Gram-stained slides. An enzyme-linked immunosorbent assay (ELISA) analysis of cytokines interleukin (IL)-1β, IL-8, CXCL10, and tumor necrosis factor-α (TNF-α) was used to determine inflammatory state of the sample. Vaginal microbiome community types (CT) were utilized to create five equivalent slide subsets for iterative development of a Gram-stained slide scoring system with comparisons with inflammatory state based on the cytokine levels.

**Results:** Digital images of the Gram-stained slides were scored based on Gram staining and morphology of bacteria, presence of sheep epithelial cells, and immune cells. The scoring system was modified in an iterative fashion with weighting based on cytokine categorization of inflamed samples, with three of four cytokine values above the mean indicating that the sample was inflamed. The parameters in the final version of the scoring system included mature epithelial cells, Gram-negative rods, and Gram-positive diplococci indicating normal and immune cells indicating inflammation. The area under the receiver operator characteristic curve (ROC AUC) was 0.725 (ROC AUCs range between 0.5 and 1.0) with a greater area indicating higher diagnostic ability of a test with a binary outcome: inflamed or normal.

**Conclusion:** The scoring system, derived from the advanced VMB and cytokine analyses, provides a validated, practical method for quantification of Gram-stained slides that can be performed in most laboratories, increasing the potential for standardization. The training plan can assist laboratories to determine the safety of intravaginal products in their sheep studies or the methodological approach can be applied to other animal models where such data are also needed.

## Introduction

The development of safe, effective biomedical products to prevent the sexual transmission of HIV remains a priority. Given the lack of an effective vaccine, over the past 15 years, the field of HIV prevention has seen the development and advancement of other strategies, such as topical microbicides and pre-exposure prophylaxis (PrEP). Vaginal application of the antiretrovirals, contraceptives, and other preventatives is actively being pursued for HIV, STI, and pregnancy prevention. This delivery route is targeted by the novel devices and products, such as intravaginal rings (IVRs), gels, tablets, and films that provide opportunities for sustained release. Prior to clinical testing such approaches must undergo strict safety evaluation to avoid local mucosal drug toxicity, tissue injury, inflammation, and negative impact on the vaginal microbiome (VMB). The altered VMBs impact drug toxicity, metabolism, efficacy, and pharmacokinetics and are associated with poor clinical trial outcomes, the implications of which have been understudied during the preclinical phase. The safety of vaginally administered products and devices for prevention of sexually transmitted HIV-1 infection must be adequately established in the animals prior to clinical testing in humans.

Development of these products relies on the use of relevant models for preclinical testing. The current animal models used in the preclinical vaginal safety assessment studies include mice, rats, guinea pigs, rabbits, sheep, and non-human primates (NHP) (1–6). Small animals are good for initial screening studies, but the structural and anatomical features of the vaginal cavities are not similar to humans. The columnar epithelium in rabbits fails to effectively model the stratified squamous epithelium in humans and the smaller vaginal cavity size of mice, rats, guinea pigs, rabbits, and NHPs require modification of intravaginal devices for preclinical testing (5, 6). While the NHP is an excellent model for efficacy, it is associated with greater expense, limited availability, more rigorous handling requirements than sheep, and modification of vaginal devices to fit the smaller vaginal cavity. Sheep are relatively inexpensive, easy to handle and sample, and can be purchased in larger numbers, while having anatomical similarities to humans allowing for direct testing of human-sized vaginal devices placement without modification (3). Several studies have established methods and outcomes after application of several over-the-counter and experimental compounds (1, 3, 7–9). In addition to standardized histopathology of the tissues after euthanasia, these now are refined repeated measure methods for ovine vaginal irritation evaluation, such as colposcopy, tissue sampling by repeat biopsy, and high-resolution imaging with optical coherence tomography (OCT) (10).

Similar to that of other animal models, the VMB of the sheep is different than that of humans, however, a compound that causes a shift in the VMB due to effects on the vaginal environment is likely to do that in any VMB community. A change in the microbiome after use of a drug may be indicative of drug toxicity and should serve as an effective surrogate of impact on the microbiota. Current literature regarding the sheep VMB is minimal and is generally focused upon the effects of pathogenic organisms on agricultural fertility without detail on comparisons between estrus and non-estrus (11–13). The sheep vaginal model is recognized by the Food and Drug Administration (FDA), which has provided guidance for future assessment of vaginally administered HIV preventive drugs in the non-clinical models and clinical studies. The FDA Guidance for Industry for Vaginal Microbicides recommends sponsors assess a topical vaginal product for the potential to cause cervicovaginal inflammation or epithelial breakdown, such as scoring systems to quantify the extent of erythema, edema, leukocyte infiltration, and ulceration or disruption of epithelium, as well as detailed histopathologic assessments of vaginal tissue. Reference controls for irritation, such as 4% nonoxynol-9 (N9) are recommended for comparison. In addition, during the regulatory process, the FDA has provided counsel to members of our team that the impact of drug-releasing IVRs on the vaginal microbiota should be assessed in sheep studies to inform subsequent product development, consistent with the published guidance for microbicide development which encourages assessment of the effects of topical drugs on vaginal pH in humans and balance of vaginal microflora in non-clinical and clinical studies (14).

A detailed study on the normal and dysbiotic VMB as well as VMB changes and proinflammatory cytokines after intravaginal placebo and irritant applicants was performed in sheep and reported elsewhere (15). Characterization of the sheep VMB was completed utilizing both the next generation sequencing (NGS) and quantitative PCR (qPCR) methods to establish common community types (CT) under the laboratory animal medicine conditions and has been reported separately (15). The goal of this work was to relate ovine VMBs to vaginal inflammatory markers through iterative development of a simple scoring system using the Gram staining of vaginal secretions similar to Nugent scoring in the clinical samples (16); such a system could be utilized in any laboratory without the need for complex assays. Additionally, the research workflow described herein can be employed to develop similar tools and methods for other animal models.

## Materials and Methods

### Study Design

#### Animal Care and Use

This study conformed to the Guide for the Care and Use of Laboratory Animals and was completed with full Institutional Animal Care and Use Committee (IACUC) approval at the Sinclair Research Center (SRC). All the animals were provided continuous veterinary care, unlimited access to feed and water, and were treated humanely. They were observed daily throughout the studies and were housed in an open-air shelter exposed to the natural light-dark cycles in June/July 2016. Non-pregnant Dorset crossbred ewes (*n* = 34) were randomized to receive 5 ml intravaginal application of a reference standard (4% N9 contraceptive gel, *n* = 10), a known chemical vaginal irritant (0.2% benzalkonium chloride, BZK, *n* = 10), a standard gel vehicle control [hydroxethyl cellulose (HEC) gel, *n* = 10], or no application (sham, *n* = 4) for 10 consecutive days (Main Period). Four of the 10 sheep in each application group, but none in the sham group were followed through a non-treated Recovery Period for an additional 11 days.

#### Sheep Vaginal Sample Collection

Periodically during the Main and Recovery Periods (Main: Days 1, 2, 3, 5, 7, and 9; Recovery: Days 11, 13, 15, 17, and 21), the sterile calcium alginate swab samples of the vaginal wall were collected from each ewe. Vaginal swab fluid (10 μl) from each time point was used to create a smear on a glass microscope slide, heat fixed, and then, subjected to standard Gram staining (BD BBL Gram Stain kit with Stabilized Iodine, Thermo Fisher Scientific, MA, USA) completed in batches. The slides were labeled and stored in the boxes at room temperature until shipped to the University of Texas Medical Branch (UTMB).

### Cytokine Analysis

The Methods for specific cytokine analysis in the sheep vagina were described by our group and employed (17) with adaptation for automated processing on the archived fluids from vaginal swabs to establish useful standards for signs of inflammation in the ovine vaginal tract. The details are reported in a separate manuscript (15). Specifically, the enzyme-linked immunosorbent assays (ELISAs) were performed to quantify the concentrations of the following cytokines: (interleukin) IL-1β, IL-6, IL-8, IL-17α, CXCL10 (aka IP-10), and tumor necrosis factor-α (TNF-α) in solutions extracted from the sheep vaginal swab samples. Kinetic vaginal sampling produced a dataset (*n* > 230 vaginal swab fluids) of natural and induced VMB dysbiosis and eubiosis to power the analyses. The ovine vaginal samples were evaluated for IL-1β, IL-6, IL-8, IL-17α, CXCL10, and TNF-α cytokine levels using the previously published methods (17) and are reported in a separate manuscript (15). These cytokines are shown previously to be the markers of vaginal inflammation and have been detected in sheep (17–19). Similarly, the established VMB CTs are reported elsewhere (15). These results were utilized to stratify the samples into equivalently distributed subsets for the iterative development of the Gram stain slide scoring system.

### Development of the Ovine Gram Stain Scoring System

**Figure 1:**
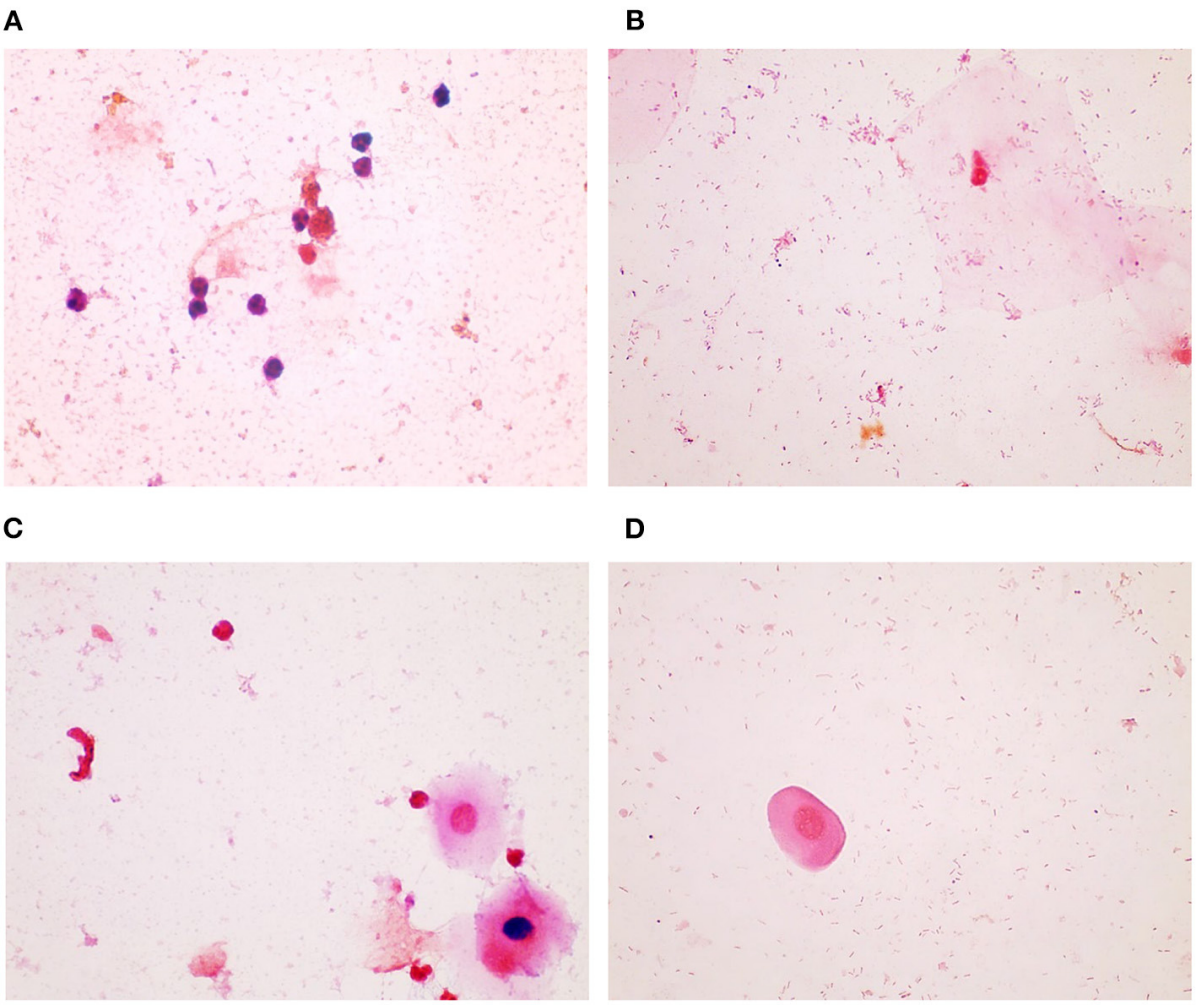
Sheep inflammatory and epithelial cells. **(A)** Inflammatory cells can be seen in this Gram-stained slide as small cells with large or multi-lobulated nuclei. The epithelial cells were seen as **(B)** mature, which are the superficial cells with small nuclei and large cytoplasm, as **(C)** intermediate, which have less cytoplasm and slightly larger nuclei, and as **(D)**, parabasal, which are near the basal or deepest cell layer and that have large nuclei and small amount of cytoplasm (micrographs are ×100 oil immersion).

**Figure 2:**
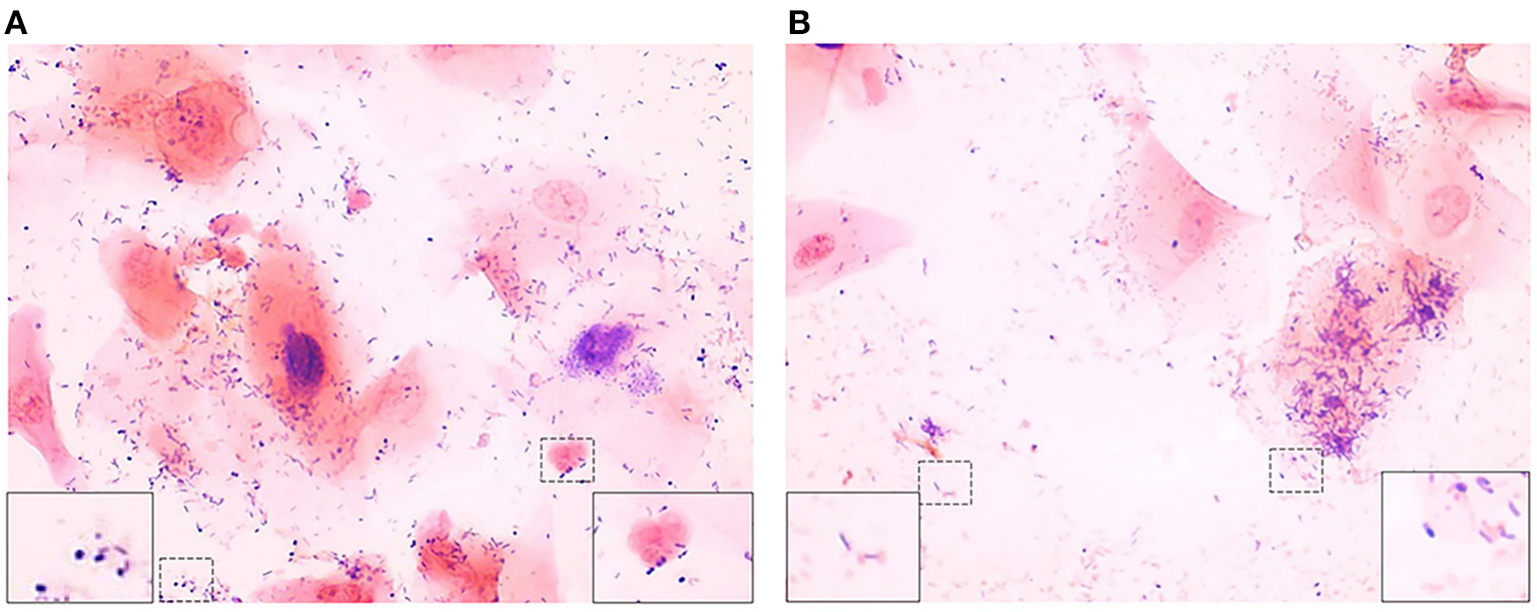
Gram-positive bacteria seen in Gram-stained slides of sheep vaginal secretions. **(A)** Gram-positive cocci (left dashed box and magnified in insert on left) and Gram-positive diplococci (right dashed box and magnified in insert on right), **(B)** Gram-positive rods (left dashed box and magnified in insert on left) and pleiomorphic appearance of Gram-positive bacteria (right dashed box and magnified in insert on right). There was poor predictive ability for Gram-positive cocci, rods, and pleiomorphic bacteria, therefore they were not included in the final reduced scoring system (micrographs are ×100 oil immersion).

**Figure 3:**
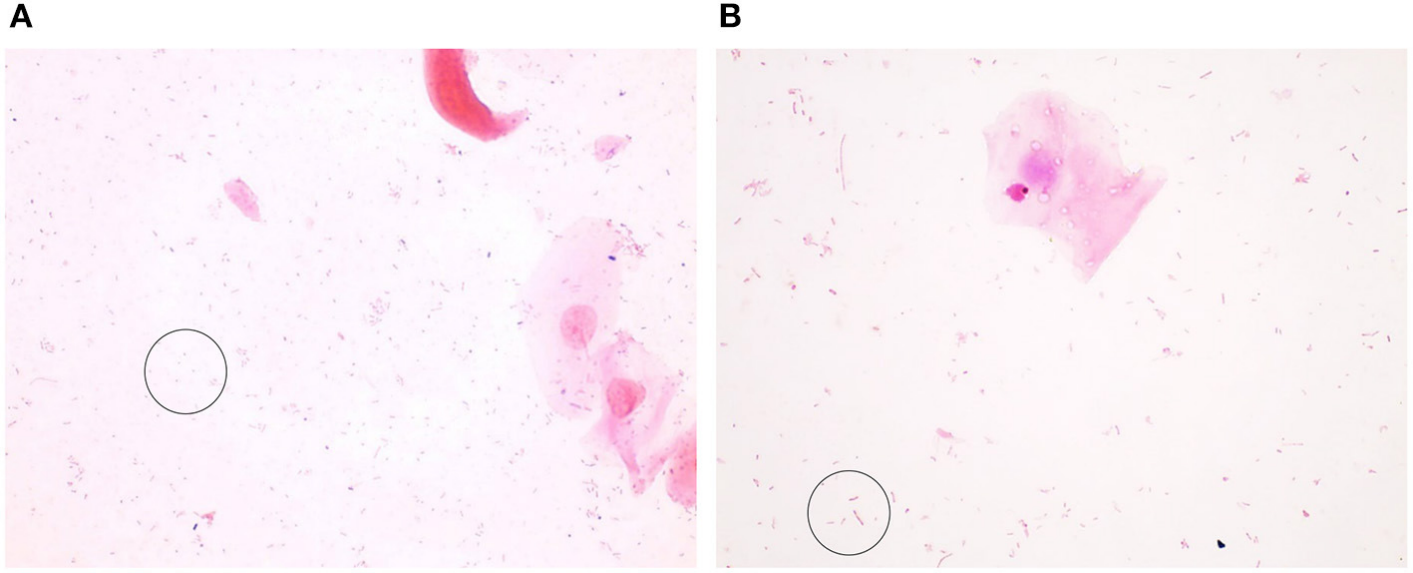
Gram-negative bacteria seen in Gram-stained slides of sheep vaginal secretions. **(A)** Gram–negative cocci (within circle) were very difficult to visualize due to pale staining and potential for confusion with debris. There was not good concordance between the graders, and this was not included in the final scoring system. **(B)** Gram–negative rods (within circle) were associated with a normal, non-inflammatory state (micrographs are ×100 oil immersion).

The Gram stains of slides made from each of the vaginal swabs were prepared and analyzed that include digital imaging and manual morphotype enumeration to support the development of an approachable scoring system to predict inflammation status. The Gram-stained slides representing all sheep and study visits were assigned to one of five subsets (designated subsamples A–E) based on VMB CT grouping to ensure a statistically similar distribution. The details of the ovine VMB and established CTs are reported elsewhere (15). Each Gram-stained slide was digitally imaged to produce 2–5 representative fields that were included in each subsample. The images in the first group (Subsample A) were reviewed to produce Gram stain morphotype and ovine cell counts that were then used to develop the initial scoring system formulas. The categories counted included the inflammatory cells and sheep epithelial cells (mature, intermediate, and immature/parabasal; Figure 1), Gram-positive (Figure 2), and Gram-negative bacteria (Figure 3), such as cocci, diplococci, rods (long and short), and the presence of mucus.

Based on the cytokine results, each sheep sample was categorized as inflamed or normal to establish the appropriate weight and association for the Gram stain morphotypes and cell type categories. The initial and then subsequent scoring system algorithms were developed by an iterative process with comparisons to inflammatory state followed by the statistical correlations and predictive value calculations to help identify the necessary refinements to the weights and values for each category. As a new scoring matrix was tested, past and current subsample data were subjected to the scoring method and re-tested for the improvements in predictive value (ROC AUC, area under the receiver operator characteristic curve). Each iteration was then tested prospectively by advancing to the next subsample, i.e., Subsamples (B–E).

#### Determination of Inflammatory Status of Samples

In addition, the inflammation status of each sample based on the cytokine profiles was considered. Several models were developed to establish if a sample was inflamed that initially involved tertile (low/medium/high; L/M/H) categorical designations but later only binary (low/high; L/H) groupings for each cytokine (Table 1). Inflamed status was equally distributed across the subsamples.

**Table 1:** Final inflammation models based on cytokine interleukin (IL)-1β, IL-8, CXCL10, and tumor necrosis factor-α (TNF-α) optical density (OD) results in tertile or binary categories.

**Inflammation model**	**OD grouping**	**Model description**
Infl1^*^	Binary low/high	3 of 4 high
Infl2	Tertile low/med/high	2 of 4 high
Infl3	Tertile low/med/high	IL-8 and IL1β only; 1 med or high
Infl4	Binary low/high	IL-8 and IL1β only; 1 high

* *Infl1 was initially evaluated using tertiles*.

#### Creation of Subsets of Slide Images Based on CT

After inventorying the Gram-stained slides, 2–5 brightfield digital images (×100 oil immersion) were collected to represent an average field for each sample. Each image was assigned a unique four-digit identifier by the EVOS imaging system that was used with standard settings for all the collections. The 264 samples (slides) and associated digital images were assigned to one of five subsamples, labeled A through E, that were designed to be statistically equivalent datasets. The subsamples were stratified by VMB CT. This process divided the sampling frame into non-overlapping subgroups, or strata. The samples were then randomly selected, without replacement, using a seed number from within each stratum and assigned to a subsample. The size of the five subsamples was predetermined as A = 53, B = 53, C = 53, D = 53, and E = 52. The asymptotic, two sample, Kolmogorov–Smirnov test was used to compare the distribution of community profiles across the subsamples confirming an equal and indistinguishable distribution. The analysis confirmed appropriate distribution of the selected samples such that subsample A effectively represented the complete dataset. Similar analyses were completed for the subsamples B–E without significant differences. All the Gram-stained slides met minimum requirements for staining intensity (empirically determined with standard settings on the EVOS microscope system) and presence of useful fields (at least three) for the analyses.

#### Scoring of Findings on Gram-Stained Slides

Based on the VMB profiles and selected dominant organisms, the Gram-stained images from various literature and web-based sources were compiled to educate operator technicians on proper identification of Gram status and morphotype. For subsample A, a single operator (Operator 1) was tasked with counting the Gram-positive and Gram-negative bacteria, such as cocci, diplococci, and rods (long and short) morphotypes as well as sheep epithelial cells (mature, intermediate, and immature), inflammatory cells, and mucus (a four-point scale for amount observed with 0 being none and 3 being large amounts). For subsample B, two trained operators were tasked with similar counts with a few refinements based on the observations in subsample A. The subsamples C–E utilized refined scoring systems where criteria for numbers of observed morphotypes and cells resulted in a score rather than a count. For these subsamples, two operators each independently scored the images. Each categorical outcome was compiled in a spread sheet under appropriate column headers. Digital images that were deemed blurry, contained too few bacteria or sheep cells or were duplicates of another image were excluded from the analyses by the independent operator without bias from other outcomes. All the operators were blinded to the inflammation status of the sample.

#### Optimization of Gram Stain Scoring System

**Figure 4:**
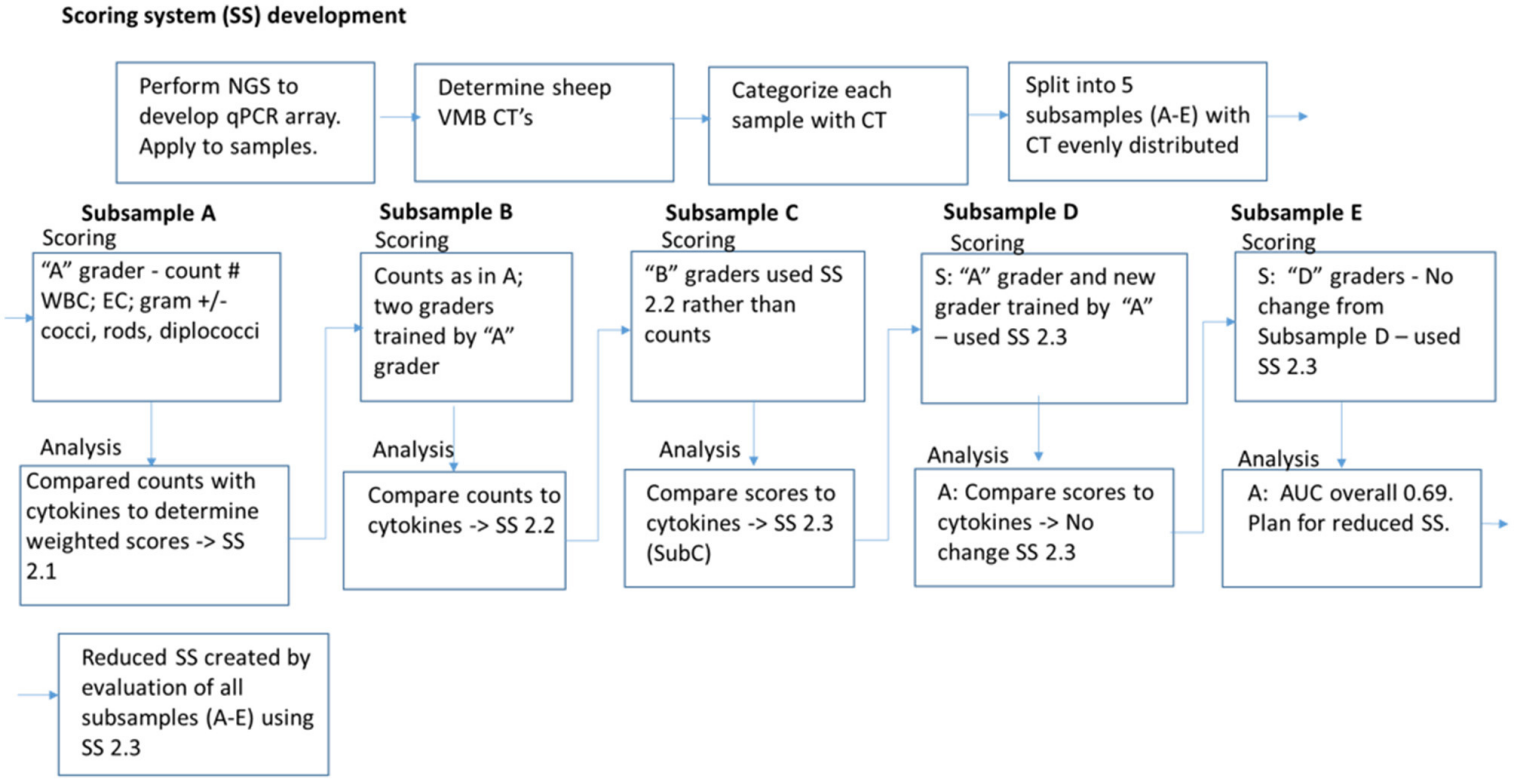
Iterative development of Gram stain slide scoring system. Vaginal microbiomes for each sample were determined by next generation sequencing (NGS) and quantitated PCR (qPCR), were categorized into community types (CT). Gram-stained slide images were split into five subsamples (A–E) with equivalent distribution of CTs. A scoring system (SS) was created based on counting the white blood cells (WBC), epithelial cells (EC), and Gram staining and morphotypes of bacteria, and applied to Subsample A. The counts were compared with cytokine-based inflamed categorization for weighting and modification of the SS. The modified SS was utilized for scoring Subsample B, and a similar process of comparison to inflammation, weighting, and modification was applied for each subsequent Subsample. After all the subsamples were analyzed with the modified SS used for Subsample E, and noted to have area under the curve (AUC) 0.69, the SS was simplified and reduced with removal of the conflicting parameters. The final reduced SS had AUC 0.725.

Using the results from each subsample, the scoring algorithms were created and refined as indicated in Figure 4. Beginning with subsample A, the counts of specific morphotypes and cell numbers were compiled for 172 digital images. Optical density values for each of the four useful cytokines were utilized to categorize each sample as inflamed (Y) or normal/not inflamed (N) based on three of four OD-values in the medium or high tertiles. The data were sorted into a set of inflamed results (*n* = 99 images) and a set of non-inflamed (*n* = 72 images) to create average and SD values for Student's *T*-test comparisons. This analysis was employed to identify the meaningful information to use for the scoring system development. The weighted score point values were assigned based on the *t*-test probability (*p*) value to create the v1.0 scoring system; the weighted scores were based on the size of the *p*-value with the smallest *p*-values assigned a larger point score to reflect the statistical likelihood that this difference was important.

Using the excel “IF:THEN” function, a scoring algorithm was applied to the counts for each of the identified categories that were significant to convert the data to a total score designed as a minimum of 0 meaning not inflamed and a 10 as most inflamed. Finally, as a first pass analysis, the concordance between the score and the inflamed categorical (Y/N based on three of four cytokines in the medium or high tertiles) to provide a percentage of correct binary concordance calls based on a score cut-off of >4 equal to abnormal as is the case for the Nugent scoring system. These data were compiled for each category and the empiric adjustments were made to create additional scoring matrices.

Each scoring system iteration was statistically evaluated for subsample A. The results of the concordance rates and the ROC AUC analyses helped to identify the optimal scoring system that was then applied to counts created by the two independent operators for subsample B. These two operators were trained with a refined set of images collected from subsample A as guidance tools. The system was further refined to create scoring matrix v2-c that was then applied as a score rather than a count to the images in subsample C by the same two operators who scored subsample B. Additional images were identified to enhance the training materials. The subsamples D and E utilized a final scoring matrix but was executed by one newly trained and the original subset “A” operator to test the validity and utility of the scoring system.

#### Training Set

A training set power point was created with representative images of each of the findings to be scored within the scoring system. This training set was created to train three additional graders, two with experience in Nugent scoring and one without scoring experience, to recognize the features included in the scoring system.

### Data Analysis

#### Cytokine Analysis

Analysis of the sheep vaginal cytokine levels, such as the significance of relationships, residuals, and outliers, was performed. A linear regression and curve fitting allowed the measured OD-values from an ELISA analysis to be used as the indicators of cytokine abundance in each of the samples.

#### Assignment to CT

Community types assignment was used to create the analytical subsamples of equal distribution to allow the development of a scoring system for the Gram-stained slide samples. In addition, normalized genomic counts were compared using a non-centered clustering algorithm (15). This clustering analysis was used to synthesize sheep VMB CT leading to the identification of biomarker, dominant bacteria community members.

#### Development of Gram-Stained Scoring System

To develop and optimize the Gram-stained slide scoring system, multiple statistical analyses were performed to interpret the inter-correlations of the sheep inflammatory and Gram stain outcomes.

After subset A was scored using the initial scoring system algorithm, Student's *T*-test comparisons were used for each bacterial morphotype or ovine cell type. Seven categories were found to be significantly different between the inflamed and normal groups. These results directed assignment of weighted scores to these seven categories based on the *p*-value. A principal component analysis (PCA) was utilized to determine the importance of different categories of the scoring system algorithm. The maximum likelihood values from a multivariate logistic regression (LR) were established for each scoring category to suggest the possible changes in weighting of the scores as well as confirmation of the direction (positive or negative) of the relationship between each score and the outcome (inflammation or normal). A LR analysis produced an ROC AUC and a Youden cut-off was calculated to estimate the score above which it would indicate inflammation for each iteration of the scoring system, with the goal of an ROC AUC of over 0.7 (20). These techniques were employed in an iterative process for each subset of images as the scoring system was optimized.

## Results

### Development of Scoring System for Gram-Stained Slides

A total of 264 slides were evaluated and digitally imaged (×100 oil immersion bright field) creating 913 digital files of representative fields (2–5 per slide). All the slides were found to be of sufficient quality for inclusion and imaging, however, during analysis, the labeling issues were discovered leading to exclusion of two slides that could not be confidently identified. During counting/scoring, some images were deemed to be of poor quality (e.g., blurred image, too few bacteria, and too high density) or were found to be duplicate images of the same field/region and were excluded by the operator.

#### Subsample A

Subsample A images (*n* = 173 total with two exclusions) were evaluated by a single operator (Operator 1) who enumerated bacterial morphotype categories (Gram-positive cocci, diplococci, rods and Gram-negative long rods, short rods, and cocci) and ovine cell type categories (mature, intermediate, parabasal epithelial cells, and immune cells). The amount of mucus present was graded as none through large amounts on a 0–4 scale. Finally, those images that contained scant numbers of bacteria were also counted as a “1.” The inflamed state of each sample was added (inflamed = 3 of 4 cytokines with tertile categories of medium and high) and the data from the 170 images were sorted into the inflamed or normal categories allowing the average values to be calculated for each category as shown in Table 2.

**Table 2:** Subsample A average counts by morphotype or cell type to establish a preliminary scoring system.

	**G+ cocci**	**G+ rods**	**G+ diplo-cocci**	**Pleio-morphic stain**	**G– cocci**	**G– rods short**	**G– rods long**	**Infl cells**	**Mat EC**	**Int EC**	**PB** **EC**	**Mucus** **0 none, 1small,** **2 mod,** **3 large**	**Scant bacteria** **0 no** **1 yes**
**INFLAMED**													
**AVG**	5.4	3.6	0.4	0.6	17.3	8.3	0.5	2.0	0.4	0.4	0.2	1.5	1.0
**SDEV**	9.1	7.0	1.1	2.7	10.8	8.1	1.4	2.8	1.2	0.8	0.5	0.6	0.1
**COUNT**	98	98	99	99	98	97	98	98	98	98	98	39	50
**NORMAL**													
**AVG**	8.8	7.6	1.5	1.0	15.0	16.7	1.9	0.1	0.8	0.3	0.1	1.8	1.0
**SDEV**	9.3	11.0	2.4	2.4	9.1	12.3	2.9	0.3	1.2	0.6	0.6	0.9	0.0
**COUNT**	72	72	70	72	72	72	72	72	72	72	72	12	25
***T*****-Test** ***P*****-value**	1.8E-2	7.2E-3	8.3E-4	4.3E-1	1.4E-1	1.6E-6	2.7E-4	5.7E-10	3.2E-2	3.1E-1	4.9E-1	3.9E-1	3.2E-1
	**Eub**	**Eub**	**Eub**			**Eub**	**Eub**	**Dys**	**Eub**				
**Score system V1.0**	**<5 = 1**	**<4 = 1**	**<1 = 1**			**<10 = 2**	**< 1 = 2**	**>1 = 3**	**<1 = 1**				

*The values from categorized digital images were sorted by inflammation status of the sample and then averaged as shown above. Student's T-test p-values were calculated with significant values highlighted in yellow indicating the bacterial morphotypes and ovine cell types that were significantly different between the two cohorts. G+, Gram-positive; G–, Gram-negative; Infl, inflammatory; Mat, mature; EC, epithelial cells; Int, intermediate; PB, parabasal; mod, moderate; AVG, average; SDEV, standard deviation; Eub, eubiotic; Dys, dysbiotic. Green highlights indicate eubiotic state while red highlights indicate dysbiotic state. Yellow highlights indicate significant difference between inflamed and normal samples in different scoring categories*.

Of the total, 99 images were categorized as derived from an inflamed sample while 72 images were from the normal samples. The number of observations for each category was calculated illustrating that mucus was present in less than half of the samples for each inflammation status (39% of inflamed and 17% of normal). Similarly, scant bacteria were observed more often in the inflamed samples than normal and was noted in 50% of those images.

Using the Student's *t*-test comparisons for each bacterial morphotype or ovine cell type, seven categories were found to be significantly different between the inflamed and normal groups. Specifically, the presence of immune cells was significantly higher in the inflamed (an average of 2 per field in inflamed and only 0.1 in normal) samples (*p* < 0.001). The presence of Gram-negative rods was significantly higher in the normal samples (average of 16.7 short and 1.9 long in normal compared with 8.3 and 0.5 in inflamed with *p* < 0.001). Gram-positive cocci, rods, and diplococci were significantly more common in the normal samples (Table 2). Finally, ovine mature epithelia were significantly more common in the normal samples (two-fold). These results directed assignment of weighted scores to these seven categories based on the *p*-value. For some of the subsequent algorithms, a score for Gram-negative cocci was included because this morphotype was predicted by the CT evaluations. As shown in the bottom row of Table 2, a v1.0 of the scoring system was established and applied to the counts to test the concordance of the total score to inflamed status. For this analysis, a score of 6 or higher was expected to be inflamed (Y) while a score of 5 or lower would be normal (N). As shown in Table 3, the v1.0 scoring algorithm was empirically adjusted by changing cut-offs for bacterial morphotype and cell counts as well as weighting for the seven categories leading to the changes in concordance rates (Table 4).

**Table 3:** Scoring system algorithms.

**Scoring algorithm**	**G+ cocci**	**G+ rods**	**G+ diplo-cocci**	**G- cocci**	**G- rods short**	**G- rods long**	**Infl cells**	**Mat. EC**	**Possible total score#**
v1.0	<5 = 1^*^	<4 = 1	<1 = 1	∧	<10 = 2	<1 = 2	>1 = 3	<1 = 1	11
v2.1	<7 = 1	<4 = 1	<1 = 1	<1 = 1	<15 = 1	<2 = 1	>0 = 3	<1 = 1	10
v3.1	<7 = 1	<4 = 1	<1 = 1	<1 = 1	<15 = 1	<2 = 1	>0 = 2	<1 = 1	9
v4	<7 = 1	<4 = 1	<1 = 1	<1 = 1	<15 = 1	<2 = 1	>0 = 2	<1 = 1	9
v2-c	<7 = 1	<4 = 1	<1 = 1	<1 = 1	<15 = 1	<2 = 1	>0 = 3	<1 = 1	10
v2.2	<7 = 1	<4 = 0	<1 = 1	<1 = 2	<15 = 1	<2 = 1	>0 = 3	<1 = 1	10
vSubC	>7 = 1	>4 = 1	0–1 = 1	0 = 1	<15 = 2		>1 = 3	0 = 1	10
Expanded								
Final reduced	**–**	**–**	**0–1 = 1**	**–**	**<15 = 2**		**>1 = 3**	**0 = 1**	7

*G+, Gram-positive; G-, Gram-negative; Infl, inflammatory; Mat, mature; EC, epithelial cells. The final Reduced scoring algorithm removed Gram-positive cocci and rods and Gram-negative cocci due to the poor predictive value of these categories*.

# *Total score is determined by the sum of scores from each category*.

* *< 5 = 1— <5 Gram-positive cocci would give a score of 1*.

∧ *Gram–negative cocci were not included in the first version 1.0 of the scoring system algorithm*.

**Table 4:** Concordance rates between total score and initial inflammation status (Infl1 and tertile) for selected scoring algorithms for initial modification (Subsample A to Subsample B).

**SubSample**	**Scoring algorithm**	**Operator**	**# Concordant**	**# Discordant**	**Total**	**% Concordance**
A	v1.0	1	132	39	171	77
A	v2.1	1	138	33	171	81
A	v3.1	1	136	35	171	80
A	v4	1	136	35	171	80
A	v2-c	1	130	41	171	76
B	v2.1	2	115	54	169	68
B	v2.1	3	128	40	168	76
B	v2.2	2	122	47	169	72
B	v2.2	3	129	39	168	77
A	v2.2	1	144	27	171	81

**Figure 5:**
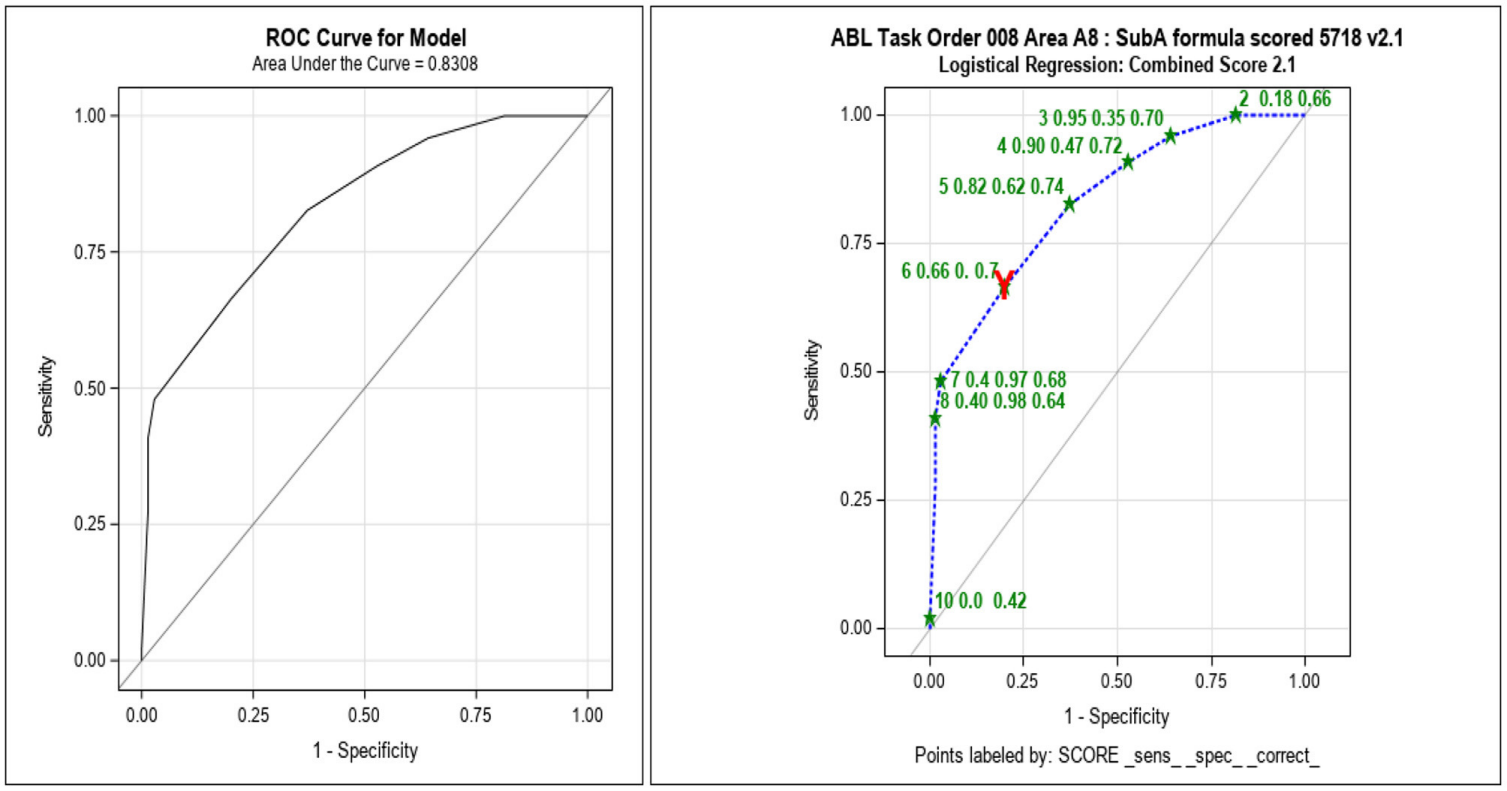
Subsample A receiver operating curve (ROC) area under the curve (AUC) analyses with v2.1 scoring algorithm showing acceptable sensitivity and specificity. After each subsample was scored and compared with inflammation as determined by cytokine analysis, an ROC AUC was calculated to determine the optimal scoring algorithm to be used for evaluation of the next subsample (_sens_, sensitivity; _spec_, specificity; _correct, proportion correctly categorized as “inflamed” or “normal,” at each cut-off). The red Y indicates the Youden, optimized, and cut-off.

Initial concordance evaluations suggested the v2.1 scoring algorithm provided the highest concordance rate (81%; Table 4). Further statistical evaluations included principal component analyses (PCA) that illustrated both the inflamed cells and Gram-negative cocci were distinct from the other categories in the response. An LR analysis produced an ROC AUC and a Youden cut-off to estimate the predictive value of the scoring algorithm (Figure 5). These analyses provided an ROC AUC of 0.83 and a Youden cut-off of 6 or higher being an indicator of inflammation.

#### Subsample B

Based on these compiled outcomes, the v2.1 algorithm was utilized without modification for evaluation of the counts for the subsample B image set. These counts were compiled by two newly trained operators (Operators 2 and 3) who were instructed to enumerate the bacterial morphotypes and ovine cell types in the eight categories shown in Table 3 for a total of 178 images. Using the modified v2.2 scoring algorithm produced better concordance outcomes for the subsample B data than v2.1 and so was also applied to the counts in subsample A (Table 4), where an equivalent concordance rate was observed. An ROC AUC analysis for Operator 2 was 0.72 and 0.79 for Operator 3. A PCA completed on subsample B confirmed the importance of immune cell presence and the absence of Gram-negative cocci as the important predictors of inflammation. Full analysis did not reveal any needed changes in the v2.2 scoring algorithm. Outliers suggested that some of the discordance may have resulted from the designating samples as inflamed based on the medium/high tertile system.

#### Subsample C

The v2.2 success supported the transition from actual counts to assigned scores to reduce labor and to begin testing the real-world application of the scoring system. The v2.2 algorithm was modified by combining the Gram-negative long and short rods into one category and changing direction of Gram-positive cocci and rods, then applied to the digital images of Gram-stained slides compiled in subsample C, leading to the algorithm designated vSubC scoring (Table 3). The trained operators (Operators 2 and 3) again independently evaluated each image. Due to a lack of published data and a potentially overly conservative categorization, the inflammation designations for the individual samples were reconsidered leading to the development of four models for converting the cytokine results to inflammation status (Table 1). The tertile system was also modified to employ a binary low/high (50th percentile) categorization for the OD-values.

A subsample C analysis included a total of 170 images and supported the Infl1 and Infl4 models that utilized the binary categorization of ELISA OD-values using all the four cytokines (Infl1) or only IL-8/IL-1β (Infl4) for designation of inflamed status. In addition, these data suggested the vSubC scoring algorithm produced consistent outcomes based on the weighting and outlier analyses. The outlier results as well as the LR analyses for each scoring category again suggested issues may exist with the values associated with Gram-positive morphotypes and that these categories may be unreliable for predictive value adding to noise in the assay. An outlier analysis suggested the discordance was not based on the technical issues (inaccurate scoring, disparate labels, etc.) but rather biologic variation expected in this system.

#### Subsample D

Based on the findings from subsample C analyses, the vSubC scoring system (Table 3) was then utilized by two operators that included a newly trained individual (Operator 4) who had Nugent scoring experience, and the initial scorer for subsample A (Operator 1). This training opportunity allowed testing of the refined training materials and approach. Both the operators completed scoring of the subsample D images independently and in blinded fashion, scoring 178 images in subsample D. The concordance rates were lower than that seen in subsample A–C, however the average scores of the operators for the datasets were identical (Table 5). The ROC AUC values were also lower than the past subsamples falling below the acceptable levels for good prediction validation. Operator 4 had values of 0.64 (Infl1) and 0.6 (Infl4) while Operator 1 had 0.62 (Infl1) and 0.59 (Infl4). The data analyses again suggested that the Gram-positive scores were adding noise and reducing predictive value of the scoring algorithm. In addition, the score values that were most predictive were the presence of immune and mature epithelial cells and the numbers of Gram-negative rods. After reviewing the data, no changes to the scoring system or selected inflammation designations (Infl1 and Infl4) were made for scoring of subsample E. Both the operators completed post-scoring re-training to increase consistency in the categorizing images.

**Table 5:** Scoring concordance between the two operators (Subsample D).

**Label**	**Operator 4**					**Operator 1**				
	***N***	**Mean**	**SDEV**	**Min**	**Max**	***N***	**Mean**	**SDEV**	**Min**	**Max**
Inflamed	178	0.91	1.38	0	3	178	1.45	1.5	0	3
Mature EC	178	0.55	0.5	0	1	178	0.52	0.5	0	1
Gram– rods	178	1.03	1	0	2	178	1.19	0.98	0	2
Gram– cocci	178	0.67	0.47	0	1	178	0.03	0.17	0	1
Gram+ rods	178	0.14	0.35	0	1	178	0.26	0.44	0	1
Gram+ cocci	178	0.23	0.42	0	1	178	0.18	0.39	0	1
Gram+ diplo	178	0.85	0.35	0	1	178	0.75	0.43	0	1
Score	178	4.39	2.16	1	8	178	4.39	2.14	1	8

*EC, epithelial cells; diplo, diplococcic; SDEV, standard deviation*.

#### Subsample E

Subsample E (*n* = 181 images) was also scored by the Operators 1 and 4 to address the impact of experience on the outcomes, with an improvement in concordance (73–75% for Operator 3 and 66–68% for Operator 1; both with Infl1 > Infl4). This is consistent with greater experience with the vSubC scoring system but again was lower than seen in the previous subsample datasets. Comparison of the individual data from the two operators showed that the average scores were similar, however, as seen in subsample D, the Gram-negative cocci and Gram-positive cocci were both four-fold different. By using the LR analyses, the impact of the Gram-positive cocci and rods again appeared to be non-predictive while the presence of immune and mature epithelial cells was most predictive. The calculated ROC AUC for Operator 4 was 0.63 for both the inflammatory models and 0.65 (Infl1) and 0.70 (Infl4) for Operator 1.

For a real-world application of this system, 5–10 fields would be evaluated and averaged across the fields to create a single score for a sample. To address this aspect of the scoring system, the image scores for the subsample D and E datasets were averaged by sample and evaluated for concordance. In general, averaging did not reveal any substantial changes in concordance rates supporting the validity of the development design and the application of the scoring algorithms.

#### Inflammation Modeling

Over the course of the project, the inflammation modeling was also considered, and as noted above, the Infl1 and Infl4 models were considered the most relevant and accurate based on the outcomes and on clinical literature. Both the models were based on binary categorization of the four cytokines most often detected in ovine vaginal fluid. Infl1 included all four targets (TNF-α, IL-8, IL1-β, and CXCL10) while Infl4 considered only IL-1b and IL-8 (Table 1). Based on the ROC AUC analyses as well as the inflamed state designation based on all four targets, Infl1 was considered the superior system and based on our data and experience, is recommended as the standard for future studies of ovine vaginal inflammation state.

#### Final Scoring System

**Figure 6:**
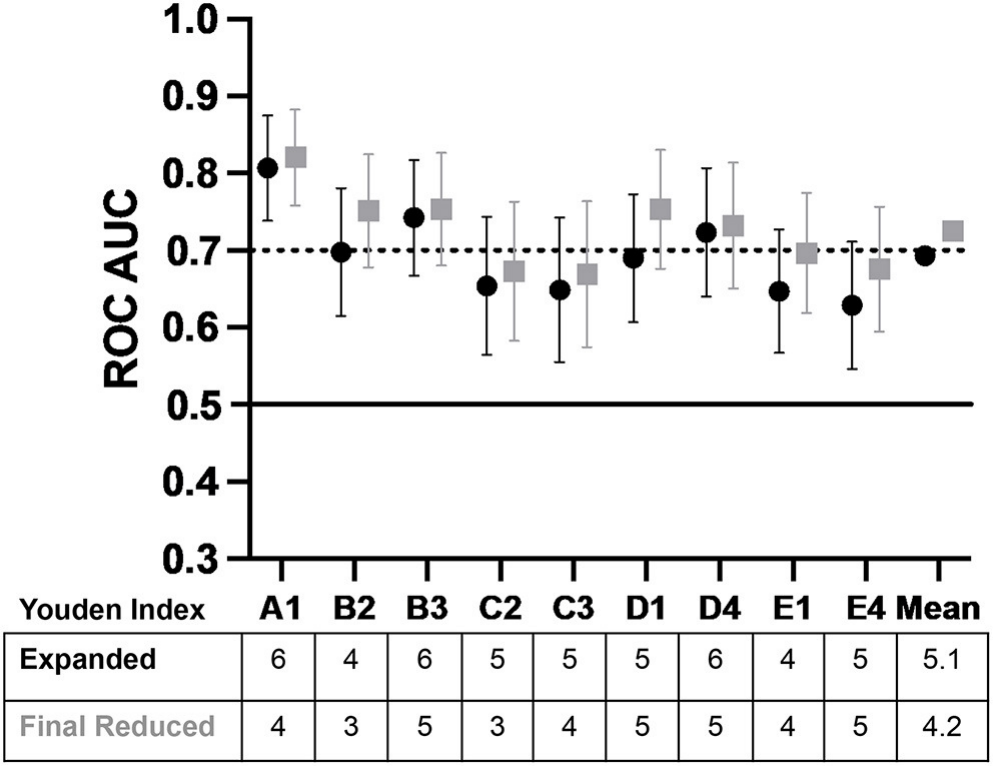
The average ROC AUC values plotted across the study showing the potential for a matured epithelial cell and immune cell, Gram-positive diplococci, and Gram-negative rod reduced scoring system. Both the final expanded and reduced systems offer acceptable predictability, however, the reduced scoring system offers a slightly higher ROC AUC (±95% *CI*). The final reduced system is simplified, scoring the most easily recognized cells and morphotypes in the current set of Gram stains supporting its utility in future work (AUC is interpreted as follows: 0.5 AUC, no predictive ability; 0.7–<0.8, acceptable predictive ability; 0.8–<0.9, excellent predictive ability). The black circles indicate final expanded scoring system which included Gram-positive cocci, Gram-positive rods, Gram-positive diplococci, Gram-negative cocci, Gram-negative rods, inflammatory cells, and mature epithelial cells. The gray squares indicate the final reduced scoring system which included Gram-positive diplococci, Gram-negative rods, inflammatory cells, and mature epithelial cells. The Youden Index, which is the cut-off above which is considered inflammation, is shown in the table under the graph, with indicators of subsample and operator (e.g., A1 is subsample A graded by Operator 1).

Overall, the results for the five subsamples indicated the vSubC scoring algorithm was consistently the most predictive, however, the average of all ROC AUC outcomes using the vSubC scoring algorithm (Figure 6; expanded scoring in black) produced a value of 0.693, just short of the goal of 0.7 for AUC. A final modification of the scoring system algorithm was performed to remove the categories that had poor predictive value. The final reduced scoring system included only the immune and mature epithelial cell categories and the Gram-positive diplococci and Gram-negative rod morphotypes (Table 3) leading to an improved overall average of 0.725 (final reduced scoring in gray; Figure 6). Both the final expanded and reduced systems offer acceptable predictability, however the final reduced scoring system offers a slightly higher ROC AUC. Additionally, the final reduced system is simplified, scoring the most easily recognized cells and morphotypes in the current set of Gram stains.

## Discussion

The overall goal of this study was to develop a method for the evaluation of Gram-stained slides to predict the presence of pro-inflammatory cytokines and dysbiosis in the ovine vagina, providing a tool that can be easily adopted by other laboratories with VMB evaluation similar to Nugent scoring in the clinical studies. The scoring system was developed in an iterative manner with the final reduced scoring system utilizing only the most correlated categories, that included ovine immune cells, mature epithelial cells, Gram-positive diplococci, and Gram-negative rods. When the full set of samples were scored using only these categories, the overall average ROC AUC was 0.725, supporting this system as a final scoring matrix for future studies. This study also provided guidance for designation of vaginal inflammation based on the presence of three of four cytokines (TNF-α, IL-8, IL1-β, and CXCL10) with values above the mean value leading to the sample categorized as inflamed. There are few data on sheep VMB, no previous reports on quantitative evaluation of Gram-stained slides and only one study in sheep reporting on the vaginal inflammatory markers (17), therefore this study provides important advancements for determining dysbiotic states after vaginal product use and enables translation from the preclinical animal safety studies to clinical studies in women.

The Gram stain scoring system for the ovine vaginal samples showed similar results between different graders, showing ease of use, and that it can be quickly taught to new operators. It was developed using the digital photographs for consistency and comparisons between graders and in an iterative manner to prevent bias and to ensure scientific rigor and reproducibility. The scoring system is intended for application to Gram-stained slides using a commonly available microscope outfitted with a ×100 oil immersion lens, therefore can be broadly utilized by many research laboratories without the need for advanced analytical methods, such as NGS, qPCR, and ELISA that were utilized in the development of the scoring system. This project also provided additional outcomes, such as methods for measuring four useful cytokines for qualifying inflammation status in the sheep vagina as well as VMB CTs that were associated with eubiosis and dysbiotic states. Additional details of the bacterial composition of the ovine VMB and marker organisms for specific CTs are reported separately (15).

The Gram stain scoring system was modeled after Nugent scoring in that Gram-positive and -negative bacteria of different morphologies were counted and included in the scoring system. The sheep scoring system also utilized the presence of immune cells and mature epithelial cells. During inflammatory states, the immune cells are significantly increased in number, and this part of the scoring system correlated highly with inflammation based on the cytokine levels. Mature epithelial cells are naturally sloughed through typical vaginal cell turnover, however, in the inflammatory states, epithelial cell sloughing can be increased, with early loss of the mature superficial epithelium and subsequent exposure of the lumen to the intermediate or immature epithelial layers.

The overall ROC AUC indicated the final scoring system had acceptable predictive ability but was limited by the presence of outliers that created statistical noise in the prediction quality. The outliers can be presented for several reasons, such as technical and biological. To reduce the noise and identify those categories of cells and morphotypes, the correlation and averaging methods were used leading to the recognition that the presence of immune cells was the most impactful outcome leading to a highly weighted score (score = 3). The immune cells can be seen at different stages of the estrus cycle, therefore a eubiotic sample could be inappropriately categorized as inflamed with the scoring system unless other categories were included. The absence of mature epithelial cells was given a score of 1 as those cells were associated with eubiosis (biologically expected due to normal sloughing). The absence of mature epithelial indicated repair and remodeling of the mucosa consistent with inflammation leading to a score of 1 when they were absent. Initially, the counts of epithelial cells were classified as mature, intermediate, and parabasal, with the hypothesis that parabasal cell presence would indicate irritation and loss of the epithelial cell layer that may lead to inflammation. Unexpectedly, there were not a high number of samples with parabasal cells, therefore the score was not deemed to be significant for prediction of inflammation, however, the presence of mature epithelial cells was highly associated with a lack of inflammation. This could contribute to technical noise due to sampling error associated with the low frequency of mature epithelial in the specific fields/images, even though, they may have been found in other fields on the slide. This is most effectively addressed by scoring multiple fields and averaging the outcomes.

An ELISA analysis of cytokines from the sheep vaginal fluid swab samples showed that IL-17α and IL-6 were rarely seen in any sheep samples and therefore were subsequently deleted from the analysis, which left only four remaining cytokines, TNF-α, IL-8, IL1-β, and CXCL10, for determining the presence or absence of inflammation. A limitation in this study was that there were no published cut-offs for the markers of inflammation in the ovine vaginal model, therefore, four inflammatory models were also evaluated and optimized during the iterative process. Additionally, this study reports on sheep housed in an open-air shelter in Missouri in summer months, whereas the preliminary data for initial classifications of CT were obtained from sheep housed indoors throughout the year in Texas. Similar to geographic differences noted in CTs in women, the variations can occur in sheep, therefore the baseline samples and studies should be considered for validation in the studies located in substantially different housing environments or geographic locations. The iterative method described for the development of the Gram stain scoring system based on the cytokines and CTs was specific to sheep, however, the process can be applied to other species as well.

In summary, this study established optimized criteria for assessing the preclinical safety of vaginal applicants and devices with respect to predicted impact on the VMB using more sophisticated and technically advanced qPCR and ELISA. The scoring system, derived from advanced VMB and cytokine analysis, provides a validated and simple quantitative method for the assessment of Gram-stained slides that can be performed in most of the laboratories, increasing the potential for standardization across the studies and laboratories. As such a work also supported the creation of a training plan to assist the laboratories who wish to export the system for their studies to help determine the safety or toxicity of vaginally administered products. Finally, completion of this project produced a workflow that can be employed for other animal models (e.g., NHP) where such data are also needed.

## Data Availability Statement

The raw data supporting the conclusions of this article will be made available by the authors, without undue reservation.

## Ethics Statement

The animal study was reviewed and approved by Sinclair Research Center.

## Author Contributions

KV, CO'N, GS, and RP contributed to conception and design of the study. Preparation of the data and analyses and manuscript drafts were completed by RP, NR-H, and KV. AM, CM, LD, and KV analyzed the samples. RP, KV, TM, CW, CO'N, and GS provided oversight and planning of the project. All authors contributed to manuscript revision, read, and approved the submitted version.

## Funding

Funding for this project was supported through the Comprehensive Resources for HIV Microbicides and Biomedical Prevention Contract Program in the Division of AIDS at NIAID/NIH (Contracts HHSN272201000001C and HHSN272201600008I). The content is solely the responsibility of the authors and does not necessarily represent the official views of the National Institutes of Health.

## Conflict of Interest

NR-H is employed by Alpha StatConsult, LLC. GS, CO'N, and CW were employed by Advanced Bioscience Laboratories, Inc. RP and KV were paid consultants to Advanced Bioscience Laboratories, Inc., under a contract with NIH (as shown in Funding). TM is employed by Sinclair Research Center. The remaining authors declare that the research was conducted in the absence of any commercial or financial relationships that could be construed as a potential conflict of interest.

## Publisher's Note

All claims expressed in this article are solely those of the authors and do not necessarily represent those of their affiliated organizations, or those of the publisher, the editors and the reviewers. Any product that may be evaluated in this article, or claim that may be made by its manufacturer, is not guaranteed or endorsed by the publisher.
